# The Molecular Epidemiology of Chronic Aflatoxin Driven Impaired Child Growth

**DOI:** 10.1155/2013/152879

**Published:** 2013-12-19

**Authors:** Paul Craig Turner

**Affiliations:** Maryland Institute for Applied Environmental Health, School of Public Health, University of Maryland, College Park, MD 20742, USA

## Abstract

Aflatoxins are toxic secondary fungal metabolites that contaminate dietary staples in tropical regions; chronic high levels of exposure are common for many of the poorest populations. Observations in animals indicate that growth and/or food utilization are adversely affected by aflatoxins. This review highlights the development of validated exposure biomarkers and their use here to assess the role of aflatoxins in early life growth retardation. Aflatoxin exposure occurs in utero and continues in early infancy as weaning foods are introduced. Using aflatoxin-albumin exposure biomarkers, five major studies clearly demonstrate strong dose response relationships between exposure in utero and/or early infancy and growth retardation, identified by reduced birth weight and/or low HAZ and WAZ scores. The epidemiological studies include cross-sectional and longitudinal surveys, though aflatoxin reduction intervention studies are now required to further support these data and guide sustainable options to reduce the burden of exposure. The use of aflatoxin exposure biomarkers was essential in understanding the observational data reviewed and will likely be a critical monitor of the effectiveness of interventions to restrict aflatoxin exposure. Given that an estimated 4.5 billion individuals live in regions at risk of dietary contamination the public health concern cannot be over stated.

## 1. Introduction

Fungal toxins, also known as mycotoxins, are frequent contaminants of dietary staples for much of the world. These potent dietary toxins are estimated to contaminate 25% of the world's cereal crops [1] making exposure frequent among many populations. Among the hundreds of mycotoxins identified, those of major public health concern include aflatoxins produced from *Aspergillus* fungi and both the fumonisins and the trichothecenes (e.g., deoxynivalenol (DON), nivalenol, and T2-toxin) from *Fusarium *fungi. Aflatoxins and fumonisins tend to be more frequent contaminants of crops in hot and humid climates as in Central America, tropical Asia, and sub-Saharan Africa where staple foods such as maize and groundnuts (peanuts) are often contaminated. Trichothecenes tend to occur more frequently in more temperate regions including parts of Asia, Europe, and North and South America [1].

This review will focus on the toxicology of the aflatoxins, the need for the development of exposure biomarkers to improve our understanding of the etiology of aflatoxin driven chronic diseases, and specifically in this review the use of aflatoxin exposure biomarkers in revealing aflatoxins role in growth faltering in infants and young children, a role that was already well established within the veterinary and other animal literature [2–18]. The recent emergence of new exposure tools for fumonisins and DON will be briefly discussed, as these may also be important contributors to some of the overall global burden of mycotoxin driven growth impairment, though the focus here will predominantly be the aflatoxin family of mycotoxins.

**Figure 1:**
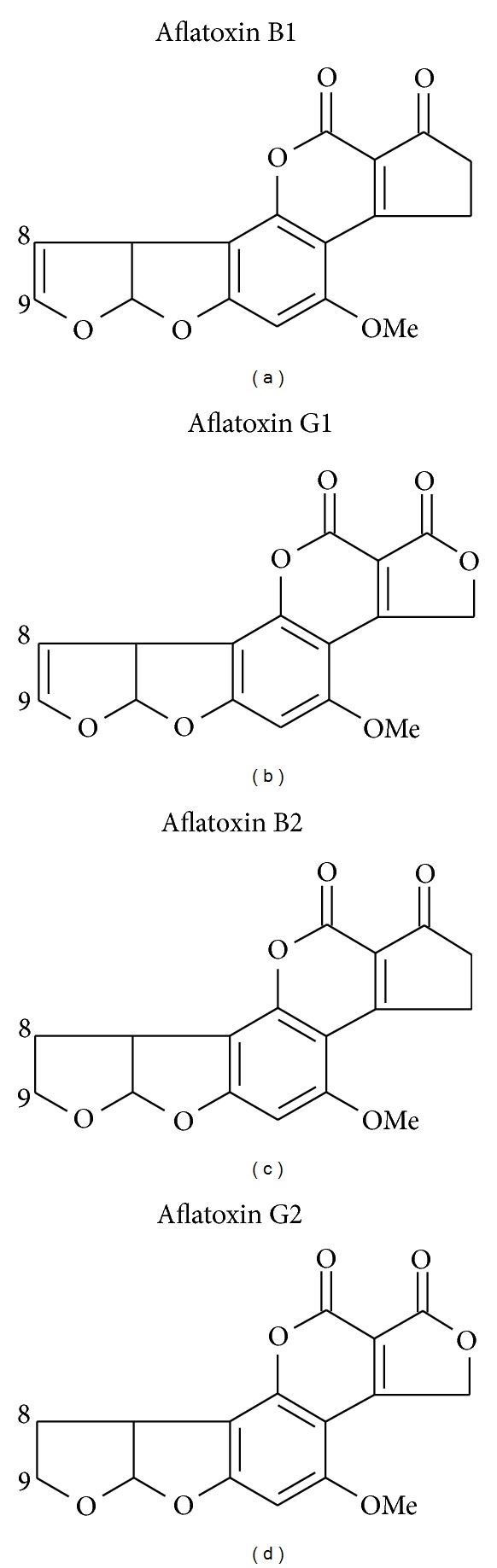
Structures of the major naturally occurring aflatoxins. Aflatoxin B1 dominates the natural occurrence and is the most toxic and carcinogenic.

The aflatoxins are a family of highly substituted coumarins containing a fused dihydrofurofuran moiety. Aflatoxin B1 (AFB1) is the most frequently occurring and the most toxic and carcinogenic, whilst other members of the family include AFB2, AFG1, and AFG2; see Figure 1. A number of *Aspergillus* strains produce aflatoxins in hot and humid climates though *A*. *flavus* (producing AFB1 and AFB2) and *A*. *parasiticus* (producing AFB1, AFB2, AFG1 and AFG2) dominate the natural production [19]. *A*. *flavus* occurs throughout the world, whilst *A*. *parasiticus* is restricted mainly to Africa, South America, Central America, and North America. For the aflatoxins both field growth and long-term storage, particularly in developing world regions, contribute to the burden of food contamination. Maize and groundnuts are two of the most frequently contaminated dietary sources in these regions and are frequently dietary staples. Aflatoxins resistance to processing and their stability during cooking also contribute to the frequency of dietary exposure [20]. Populations that are particularly likely to have chronic and high levels of exposure are typically poor, have limited dietary variety, and are reliant on maize or groundnuts as dietary staples [21, 22].

Whilst the toxicity of many mycotoxins has clearly been demonstrated in animal models the public health concerns of exposure remain poorly examined for most, in part because of a lack of useful exposure assessment tools [21–25]. Our understanding of the chronic effects of aflatoxins is perhaps the exception, driven strongly by the desire to understand their role in human carcinogenesis, and was significantly assisted by the development and validation of exposure biomarkers. Although typically recognized as human liver carcinogens, suspected for several decades and demonstrated two decades ago [26], this review will focus on a more recently emerging concern for this class of toxins. The exposure tools used to assess the role of aflatoxins in cancer have proven to be useful in this respect and their development is described below.

## 2. Mycotoxin Exposure Biomarkers

In order to better understand the role of environmental exposures in human disease, accurate and relevant exposures assessment remains a critical and complex component. Exposure to mycotoxins is frequent, but due to their heterogeneous distribution traditional nutritional epidemiological approaches are not particularly sensitive tools for exposure assessment. Additionally many world regions where populations are at risk from chronic high level exposures have monotonous diets, further weakening traditional approaches of exposure assessment. In response, exposure biomarkers offer the potential to improve exposure assessment, and these typically are measures of critical metabolites and/or the parent toxin in a biological matrix, most frequently urine or blood, though nail, hair, feces, sputum, or exfoliated cells are additional potential matrixes to consider. It is worth emphasizing that even well validated exposure biomarkers do not provide an absolute assessment of exposure but rather they offer “improved exposure” assessment. It is also important to note that a simple ability to accurately measure a toxin (or metabolite) in a biomatrix is not sufficient in itself within an exposure assessment need and that a demonstrated relationship between intake of the toxin and the “biomeasure” is also required. This requirement is exemplified by the studies on aflatoxin. AFB1 as the most frequently occurring of this family of toxins will in part be transferred unmetabolized to urine; however, the concentration of AFB1 itself in urine does not reflect the quantity of toxin ingested [27]; in fact, an inverse relationship was observed. Thus accurate LC-MS/MS or other instruments that measure this analyte in urine with exquisite accuracy, sensitivity, and reproducibility provide no useful information on the “amount of exposure” to a given individual, though a positive sample would indicate some level of exposure had occurred.

### 2.1. Aflatoxin Biotransformation and Aflatoxin Biomeasures

**Figure 2:**
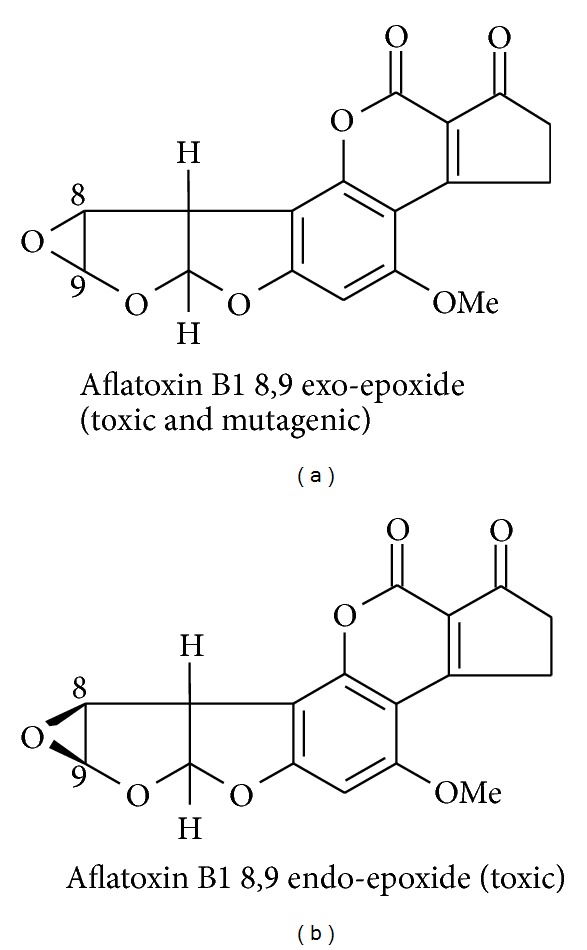
Structures of the two major aflatoxin B1 epoxides. The exo-epoxide is both toxic and carcinogenic; the endo-epoxide is only toxic.

AFB1 requires activation prior to exerting toxicity and is metabolized by a number of different enzyme systems, though several cytochrome P450 enzymes play a major role [28–30]. A series of monohydroxyl (e.g., AFM1, AFQ1, AFP1, and aflatoxicol (AFL)) and two epoxide species are recognized; AFB1-exo-8,9-epoxide and AFB-endo-8,9-epoxide are the dominant toxic metabolites; see Figure 2. Whilst most of these reactions involve an oxidative process with hydroxyl addition, AFL formation is a reductive process of the ketone moiety. In general, the hydroxyl metabolites are considered less toxic and can undergo additional phase 2 reactions involving conjugation with glucuronide and/or sulphate groups to aid excretion. The two epoxides can also undergo phase 2 detoxification reactions by binding to the tripeptide glutathione (GSH), facilitated by a family of glutathione-S-transferase enzymes, and subsequent formation of an aflatoxin-mercapturate which is readily excreted.

There are numerous other studies that have measured either AFM1 or AFB1 in serum, including studies in Nigeria, Kenya, Ghana, Sudan, Egypt, Turkey, United Arab Emirates, Argentina, Singapore, Nepal, Japan, and Thailand [31–51]. In general, areas with expected higher aflatoxin contamination of food items have higher frequencies and/or levels of these biomeasures though to date serum aflatoxin measures have not been quantitatively related to intake. Thus they remain good measures that indicate some exposure but provide limited quantitation of that risk.

Additionally a strong dose response relationship has not as yet been demonstrated between aflatoxin intake and AFM1 in breast milk; thus these are also classified here as biomeasures of maternal exposure not exposure biomarkers. Numerous studies from various global regions including Nigeria, Gambia, United Arab Emirates, Egypt, Sudan, Thailand, Turkey, Iran, and Jordan have observed aflatoxins in breast milk [37, 52–69], and as with the serum measures, higher levels tend to occur in regions predicted to be at higher risk from food contamination. Some of the above studies have used this measure in attempts to better define maternal exposure whilst others are used to indicate risk of infant exposures. For lipophilic xenobiotics in general, the measuring and understanding of the kinetics and variations of those kinetics of the transfer from maternal exposure to breast milk samples are complex [70]. For aflatoxins a small survey from Gambia (*n* = 5) over several days collection suggested that about 0.1–0.4% of the ingested AFB1 was transferred to milk [69]. A more recent survey (*n* = 50) from Nigeria has suggested a statistically significant, quantitative relationship (*r* = 0.33, *P* < 0.05) between breast milk AFM1, in a single collection sample for each person, and the amount of AFB1 in the diet [53]. Thus the variation in breast milk AFM1 concentration was poorly to modestly explained by their dietary measure. However, it remained unclear whether aflatoxin intake at an individual level (e.g., aflatoxin ng/Kg bw/day) or aflatoxin contamination levels in food (e.g., aflatoxin ug/Kg food) was compared with breast milk AFM1 concentration; thus some caution is required in data interpretation. The latter study whilst indicative of some association suggests that further evaluation is required.

Aflatoxins in breast milk where mothers are exposed remains a poorly examined potential health concern of the newborn infant. The risk is in part not only due to the infant's reliance on this nutrient source, but also because the infant is rapidly developing with poor detoxification capabilities compared to older children or adults. These concerns and solutions are discussed in detail in subsequent sections on aflatoxin interventions.

### 2.2. Aflatoxins and Exposure Biomarkers

**Figure 3:**
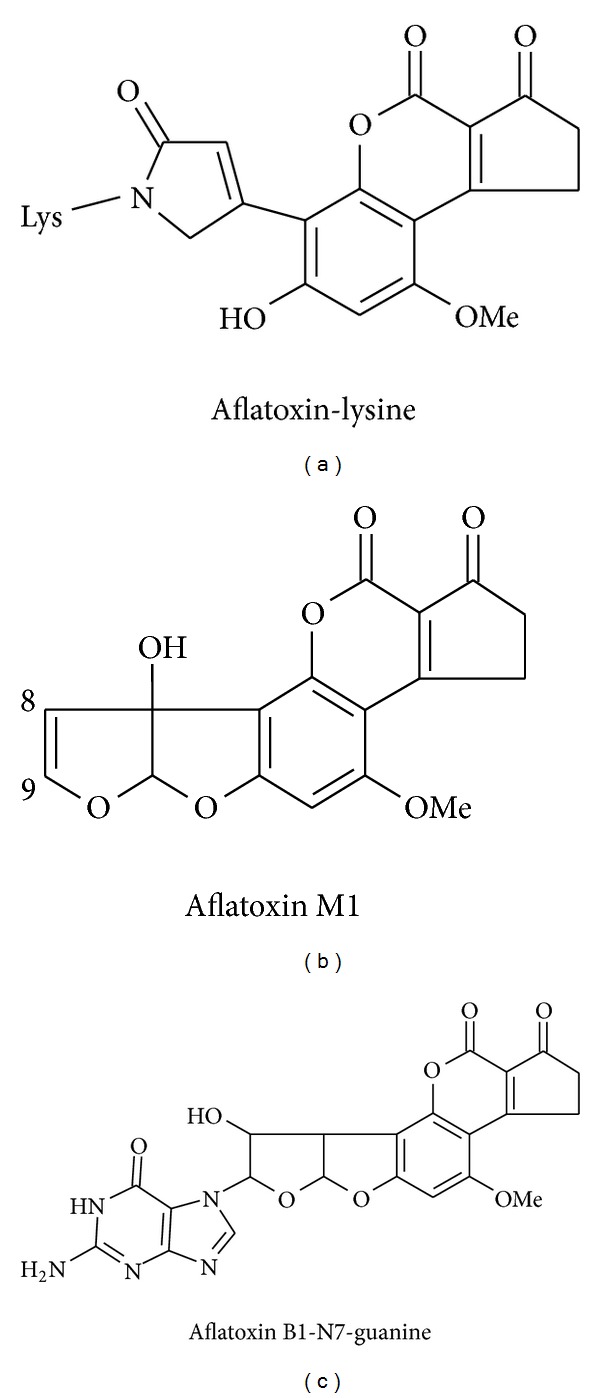
Structures of the aflatoxin species measured is validated exposure biomarkers. Aflatoxin-lysine is the digest product of aflatoxin-albumin detected in sera, AFM1 is the hydroxy metabolite detected in urine, and aflatoxin-N7-guanine is the depurination product of aflatoxin-DNA adducts.

The two aflatoxin epoxides generated by phase 1 biotransformation are highly reactive and can bind to and cause cellular and macromolecule damage [24, 28, 30, 71]. AFB1-exo-8,9 epoxide binds at the N7 position of guanine in DNA [72], and following depurination of this DNA adduct, aflatoxin-N7-guanine (AF-N7-Gua) can be transferred to urine [27, 73]. The hydroxymetabolites and the unmetabolized parent toxins can also be detected in urine [23, 24, 74]. However, in urine only the concentration of AFB1-N7-Gua (*r* = 0.80, *P* < 0.0001) and the concentration of AFM1 (*r* = 0.82, *P* < 0.0001) have been correlated with aflatoxin intake in chronically exposed individuals [27, 73, 75]. These quantitative relationships for urinary aflatoxins provide confidence in the use of these measures as exposure assessment tools, and here “urinary AFM1 and urinary AFB1-N7-Gua are defined as aflatoxin exposure biomarkers.” The structures of these exposure biomarkers are shown in Figure 3. These exposure biomarkers have more frequently been used in studies investigating the etiology of aflatoxins in hepatocellular carcinoma and are reviewed in greater detail by Kensler et al. [71]**   **and the references therein.

**Figure 4:**
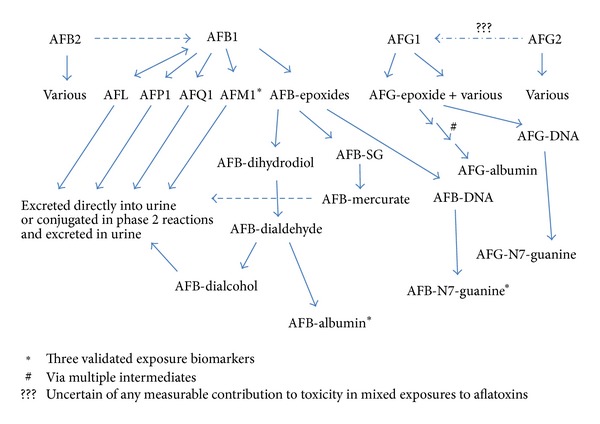
Selected biotransformation pathways for the aflatoxins. Focus on AFB1 biotransformations which are highlighted and indicate the route to the specific biomarkers. Biotransformation of AFB2 to AFB1 will be modest and likely represent less than 1% of the dose of AFB2. The AFG2 pathway is predicted, but likely of low or very modest contribution. Modified from [21, 22].

Both the endo- and the exo-epoxides of AFB1 can bind to many macromolecules in addition to DNA potentially causing their dysfunction; they can also bind at low levels to the protein albumin forming AFB-albumin, which subsequently enters the systemic circulation. AFG1 can undergo similar biotransformations to those of AFB1 including formation of reactive epoxides at the 8,9 position, and while less potent than AFB1, there is sufficient evidence that it can form an AFG-albumin adduct analogous to that of AFB1, albeit at lower levels to equivalent amounts of AFB1 [76]. AFB2 and AFG2 are not directly able to form the epoxides [77] as they lack the 8,9 double bond, though for AFB2 aflatoxin residues in macromolecules resembling those of AFB1 have been observed [77, 78]. These authors suggested that less than 1% of the AFB2 may be transformed to AFB1 in vivo and then undergo the numerous metabolic pathways of this chemical species, and therefore they likely provide negligible/modest contribution to aflatoxin-epoxide formation compared to that caused by AFB1 itself. A similar pathway for AFG2 is plausible but to date there is insufficient evidence suggestive that the AFG2 to AFG1 pathway is a significant contributor to total aflatoxin-epoxide formation in aflatoxin exposed individuals. In addition to AFB1 appearing to dominate the aflatoxins for AF-epoxide formation in experimental systems it is additionally worth remembering that, in naturally contaminated food, AFB1 is the most frequent of the aflatoxin contaminants [1, 19, 26, 79]. An overview of aflatoxin biotransformation with respect to biomarkers established is presented in Figure 4.

Aflatoxin-albumin adducts have predominantly been used in studies of child growth and are discussed in greater detail here. Albumin adduction by aflatoxin epoxides appears to occur predominantly at the free primary amine moiety of lysine residues forming a specific and stable aflatoxin-albumin (AF-albumin) product. For the purpose of clarity AF-albumin will be used unless the experimental system or analytical tool is specifically measuring AFB-albumin. AF-albumin can typically be detected if adduct levels exceed about one molecule of aflatoxin covalently bound to albumin per two million molecules of albumin in the circulation, equivalent to about 3 pg/mg albumin [80], though slightly more sensitive LC-MS/MS methods are now developed [81–84]. AF-albumin adducts are frequently observed in the sera of exposed animals and humans [85–120] and the concentration of this adduct in humans, often measured as AFB-lysine equivalents/mg albumin, was demonstrated to strongly correlate with aflatoxin intake (*r* = 0.69, *P* < 0.0001) [87, 88]. The AFB-lysine digest product of AFB-albumin is shown in Figure 3. Given the half-life of albumin, AF-albumin represents an integrated assessment of aflatoxin exposure over a period of two to three months [121]. In addition to the above correlations in human studies, AFB-albumin concentrations were demonstrated to be linear with dose in rodents across an extremely wide dosing range (0.16 ng/kg bw to 12,300 ng/kg bw; *r*_2_ = 0.98), and importantly, typical human exposures within low, moderate, and high risk communities typically fall within this experimental range [113].

In high risk regions of the world, greater than 95% of those individuals tested are positive for AF-albumin, typically over a 2-3 log range, from about 3 pg/mg albumin to approximately 1000 pg/mg (reviewed in [21]), while more developed regions rarely have detectable levels of the biomarker [21, 84, 122, 123]. Exposures in regions such as Egypt and Brazil are perhaps intermediate in exposure [64, 65, 74, 99, 112] but these exposures are still of concern. A number of approaches have been used to assess the concentration of aflatoxin exposure biomarkers including ELISA, RIA, HPLC with fluorescence (HPLC-Fl) detection, LC-MS and LC-MS/MS, and AMS; each approach has advantages and disadvantages, though readers should rather be aware that it is not always possible to compare data directly from one approach to another. For example LC-MS/MS methods provide both high specificity and accuracy to measure AFB-lysine in the assay to measure AFB-albumin; however, the processing of AFB-albumin to release AFB-lysine may not yield 100% release of the analyte; rather some incomplete digest products may also be present that contain aflatoxin residues that are not identified due to the precision of the assay for AFB-lysine specifically. In addition LC-MS/MS would not pick up AFG-lysine formed by the naturally occurring AFG1, nor the potential albumin adducts from some of the aflatoxin species (e.g., AFM1, AFQ1, and AFP1) via 8,9-epoxidation biotransformations [82, 83]. However, these latter species are not formally recognized and it is likely that they will represent negligible or no contribution compared to that of the main AFB-albumin adduct, though this has not been strictly investigated.

The frequently used ELISA method on the other hand is very accurate but may have modest cross reactivity with the incomplete “aflatoxin containing” digest products of AFB-albumin and some recognition of other aflatoxin-albumin digest products, thus, potentially provides a more integrated burden measure for total aflatoxin exposure. To compare methodologies two studies were conducted in populations in which the diet was naturally contaminated with aflatoxins. In the first a small number of sera (*n* = 20) were analyzed for AF-albumin by both ELISA and LC-MS/MS and a strong correlation between the measures was observed (*r* = 0.89, *P* < 0.0001); however, AF-albumin measured by ELISA was typically somewhat higher than AFB-albumin measured by LC-MS/MS [83]. A second survey both compared a greater number of samples (*n* = 102) and included a larger variation in adduct level (a 3 log variation), using ELISA, LC-MS/MS, and additionally HPLC-fluorescence. Similar strong correlations were observed between all three approaches, and subset analysis within the higher adduct burden group confirmed that these correlations were not simply a reflection of the wide variations in adduct levels [124]. As with the first survey the ELISA data in this second survey was somewhat higher than data generated by the other approaches. Overall these data strongly support the use of multiple methods to assess exposures but highlight that methodological comparisons should be conducted ahead of attempts to compare data from different surveys if different analytical tools are used. Yard et al. [120] recently suggested a conversion to attempt to compare the LC-MS/MS data with the ELISA data based on observations by Scholl et al. [83] and McCoy et al. [124]; though these authors were aware of the limitations, they do provide some opportunity for the purpose of comparison.

## 3. Epidemiology of Aflatoxin Driven Growth Faltering

AF-albumin biomarker driven biomonitoring within Africa reveals that a pattern of chronic aflatoxin exposure occurs in utero, during early life and childhood, and continues into adulthood (Wild et al., 1990b; Gong et al., 2012 [90, 94–96, 98, 102, 104]). In sub-Saharan Africa the variation in exposure within similar settings can span a three log difference. It is worth noting that a relatively low exposure region like sub-Saharan Africa would typically have greater levels of exposure than those occurring for developed world regions, such as Western Europe and North America [21, 84, 122, 123, 125]. In Gambia it has been suggested that whilst dietary insufficiency and infectious disease are important components of early life growth faltering, they only explain about half of the restricted growth [126–131]. These authors also reveal that intestinal enteropathy seems to parallel the growth deficits.

A series of five aflatoxin biomarker driven surveys to compare aflatoxin exposure with infant and child growth have been conducted using AF-albumin as the exposure biomarker. The distributions of AF-albumin concentrations in four of these studies are shown in Table 1. The details of the distribution for the fifth study are not known by this author. All of the AF-albumin assessments used ELISA except for study five which used HPLC with fluorescence detection.

### 3.1. Study 1

The first survey was conducted in Gambia between May 1998 and February 1999 and involved the collection of a single blood sample and anthropometric measures from 472 children aged 6–9 years [95]. AF-albumin adducts were detected in 93% of the children (geometric mean level 22 pg/mg; range 5–456 pg/mg), and AF-albumin level varied by month of sampling (*P* < 0.001). After adjustment for month of sampling, age, and sex there remained a statistically significant reduction in weight for height *Z* (WHZ) scores in relation to AF-albumin adduct level (*P* < 0.05), though no statistically significant relationship was observed between AF-albumin and weight-for-age (WAZ) or height-for-age (HAZ)* Z* scores. Of additional note was the observation that aflatoxin biomarker positivity was associated with a decrease in mean secretory IgA concentration in saliva, an important barrier that protects against infection. In this review, these were the oldest children examined for growth impairment and in this cross-sectional study recent (few months) aflatoxin exposure was only modestly associated with any growth effects.

### 3.2. Study 2

The second survey was also cross-sectional in design but recruited younger children from Benin and Togo from January 2000 to April 2000. The survey collected blood and anthropometric data from 479 children aged 9 months to 5 years. AF-albumin adducts were detected in 475/479 (99%) of the children (geometric mean adduct level 33 pg/mg, range <3 pg/mg (the detection limit)–1064 pg/mg) and revealed two notable observations [102, 103]. Firstly, for children aged 3 years and less, there was a significant increase in aflatoxin biomarker level as infants moved from being partially to fully weaned (*P* < 0.001), suggestive of some protection from exposure whilst breast feeding was maintained; that is, the weaning food becomes the major source of exposure. Secondly there were strong inverse relationships between the AF-albumin adduct levels and both HAZ (*P* < 0.001) and WAZ (*P* < 0.004), indicators of a role in stunting and being underweight. The above highly statistically significant relationships were multivariate analysis that accounted for socioeconomic status, village, and sex. Whilst observing stronger relationships between aflatoxin exposure and growth in this survey compared to the Gambian survey, it should be noted that (a) the age group is different; (b) the exposure whilst chronic in both studies was somewhat higher in the Beninese children; to date no specific aflatoxin exposure level, exposure duration, nor critical time of exposure onset has been established; (c) a dietary supplementation was taking place in the former study; and (d) the former study involved collections over two harvest seasons whilst the latter was over one and therefore less likely to be influenced by external seasonal factors that may also be important in these settings [132]. Seasonal variation in aflatoxin exposure is also known [90, 94] and was apparent in the Gambian study [95].

In a survey of Canadian sera for AF-albumin using the standard ELISA method of Chapot and Wild [80], 100% of 200 samples were below the limit of detection (3 pg/mg) by this assay (reviewed by [22]) and thus, a likely overestimate that the mean adduct level would perhaps be half the limit of detection (i.e., 1.5 pg/mg). A more comprehensive survey using samples of US residence (*n* = 2051) used LC-MS/MS (with a lower analytical sensitivity; LOD of around 0.6 pg/mg) and determined that only 1.2% were above the LOD for AFB-albumin, with a maximum of only 4.4 pg/mg. Thus overall it can be estimated that the average serum AF-albumin in this representative US survey was only 0.3 pg/mg. According to the assay comparisons of Scholl et al. [82, 83] and McCoy et al. [124], and discussed by Yard et al. [120], it is reasonable to multiply the LC-MS/MS data by 3.3 prior to comparison with the ELISA data. Thus a comparative mean of about 1 pg/mg is best estimate of the average for the NHANES data. Thus the AF-albumin burden for the high level exposures in the 6–9-year-old Gambians [95] and the 1–5-year-old Beninese [102, 103] would be in the region of 100 to 1000 times higher than that typically seen in the USA and/or Canada, a low risk world region.

### 3.3. Study 3

The third study conducted in Gambia in 2000 was longitudinal in nature and examined the role of maternal aflatoxin exposure during pregnancy, early infant aflatoxin exposure, and infant growth velocity over the first 52 weeks of life for 138 singleton births [98]. Aflatoxin exposure was assessed using AF-albumin exposure biomarkers, as before, at two time points during pregnancy (2nd and 3rd trimester and a mean obtained), in cord blood, in week 16 infant blood, and week 52 infant blood. Birth anthropometry was assessed and thereafter every four weeks until aged 52 weeks. AF-albumin was detected in all maternal samples (GM 40 pg/mg; range 5–261 pg/mg), in 49% of cord bloods (GM 10 pg/mg; range nd-190 pg/mg), in 11% of week 16 sera (GM 9 pg/mg; range nd-30 pg/mg), and in 92% of week 16 sera (GM 60 pg/mg; range nd-391 pg/mg). For the 49% of positive cord blood samples the adduct level was approximately 5–10-fold lower than that of the paired mean maternal sample, though of course samples were not collected at the same time points. This ratio was in a good agreement with an earlier study where a tighter collection time frame was used with paired cord and maternal samples [89]. The very early appearance of AF-albumin in sera samples, assessed at 16 weeks, was a particular concern and was associated with the early introduction of weaning foods, in some cases between the ages of 8 and 12 weeks. Thus aflatoxin exposure would appear to be lessened by a delay in wean food introduction. These observations are in a good agreement with the lower adduct burden in transitioning from being partially weaned to being fully weaned as occurred in young Beninese infants [102, 103]. By week 52 mothers had initiated all infants on at least some introductory weaning foods, and 92% were AF-albumin positive; many had levels typically seen in older children. Again as a comparison with the US/Canadian data some of these one-year-old Gambian infants could be estimated to have AF-albumin burdens that were one to two hundred times greater than that of individuals in the low risk region. In terms of growth velocity, AF-albumin in maternal blood was a strong inverse predictor of both weight (*P* < 0.012) and height (*P* < 0.044) gain of the infant over the first year, with lower gains in those with higher exposure. Maternal and week 16 AF-albumin combined were also significantly negatively correlated (*P* < 0.001) with growth velocity of the infant in the first year of life [98]. These data suggested that reduction in maternal AF-albumin adduct during pregnancy from 110 pg/mg to 10 pg/mg would improve linear growth in the first year of life by 2 cm and weight by 0.8 kg.

### 3.4. Study 4

The fourth study was conducted in Benin and included 200 children from four villages who were followed up for over 8 months. Recruitment was in February 2001 (age range 16–37 months), and blood samples were collected at recruitment (February) and then again in both June and October for AF-albumin analysis. AF-albumin was detected with a prevalence of 98, 99.5, and 100%, respectively, and the overall adduct range was nondetect to 1,100 pg/mg [104]. These higher levels would indicate a burden of 500 to 1000 times that of those in the US and Canadian surveys. All infants had weaning foods introduced by study entry and about 64% were regarded as being fully weaned. In each village those who were fully weaned had significantly (*P* < 0.0001) higher mean AF-albumin adduct level compared to those who were weaned at entry. Complete data at three time points was available for 181/200 (90.5%) of the children. When comparing the individual biomarker levels across the three time points there was a significant positive correlation for February versus June (*r* = 0.6253, *P* < 0.0001), for February versus October (0.5624, *P* < 0.0001), and for June versus October (0.5398, *P* < 0.0001). Overall there was a trend for higher aflatoxin adduct levels from February to October. It is not clear if this in part reflects a seasonal change in contamination, though the weaning status was also changing and more children were fully weaned in October (96%) compared to February (68%). Growth velocity of the children was assessed with respect to the AF-albumin adduct level. AF-albumin adduct level at study entry was significantly associated (*P* = 0.003) with a reduction in height increase over 8 months and additionally with mean adduct level across the 8-month period (*P* < 0.0001). Data from this study was suggestive that a 100 pg/mg difference in AF-albumin approximates to, on average, about a 1 cm reduction in height over an 8-month period in this age group. All statistical models adjusted for confounders including maternal socioeconomic status, child sex, and village.

### 3.5. Study 5

A fifth study conducted in Ghana in 2006 was a cross-sectional hospital based survey that assessed birth weight outcomes of 785 singleton births in relation to maternal aflatoxin exposure [105]. Blood sampling was close to the time of birth and given that it reflects an integrated measure of exposure to aflatoxins, this will be a strong predictor of exposures over the last few months of pregnancy. All samples were positive for AFB-albumin (mean 11 pg/mg, range 0.5–269 pg/mg). Blood samples for the first four studies and the Canadian data were all analyzed by the same method, whilst this latter study used an HPLC-fluorescence approach; thus some caution in direct comparisons is merited. However, the range of levels observed here is in rough agreement with those earlier studies, though the mean is perhaps more similar to studies in Guinea [96], another high risk country. Overall exposure seems high and frequent. A significant inverse relationship between birth weight and quartile of maternal AFB-albumin adduct level was observed. Mothers with the highest AFB-albumin quartile were more likely to have low birth weight babies (OR, 2.09; 95% CI, 1.19–3.68), with a trend of increasing risk for low birth weight (*P*_trend_ = 0.007) compared to participants in the lowest quartile [105]. All data were adjusted for sociodemographic variables and potential confounding factors.

There are several other studies of some note with respect to aflatoxin and birth outcomes with several showing significant negative correlations between birth anthropometry or child undernutrition and aflatoxin measures in biofluids [32–34, 38, 41, 42, 62, 133, 134], whilst one reported study found no statistically significant associations [135]. Importantly, this current set of studies used only “aflatoxin biomeasures” and none of these studies used validated exposure biomarker (AF-albumin in sera, AF-N7-Guanine in urine, nor AFM1 in urine) to assess the level of aflatoxin exposure; thus, quantitative relationships between the biomeasure and exposure outcome are not straightforward to interpret. However, these studies broadly support the above investigations in which validated exposure biomarkers were used [95, 98, 102–105].

Overall three messages are apparent from the above data [95, 98, 102–105]. Firstly, the presence of cord blood AF-albumin indicates transplacental transfer of aflatoxin from mother to fetus and that at some point in this exposure the fetus can activate the toxin to the reactive epoxide, with the epoxide being the reactive metabolite that is capable of causing toxicity. Maternal aflatoxin exposure was associated with reduced birth weight [105] and longitudinal growth faltering during the first year of infant life [98]. For these two specific studies, the longer term effects of maternal aflatoxin exposure and child growth past one year are unclear, and because the infants' diet will become the predominate route of continuing aflatoxin exposure, this latter route of exposure will likely increasingly dominate any continued aflatoxicosis into and throughout early and later childhood. A potential role of early life “programming” through in utero exposure is plausible but remains to be fully assessed. Also, no studies have followed infants from the in utero period past the first year of life, and this remains a gap in our knowledge. Secondly, breast feeding was associated with a lower body burden of aflatoxin biomarkers in the infants compared to those partially and fully weaned, most likely reflective of lower levels of aflatoxins in the breast milk (transferred from maternal exposure) compared to weaning foods. Thirdly, in all surveys dose dependent associations between AF-albumin adduct level and growth/growth velocity were observed. In separate studies this effect on growth was apparent whether exposure was assessed at 16 weeks [98], 9–60 months [102, 103], or 16–37 months of age [104].

**Figure 5:**
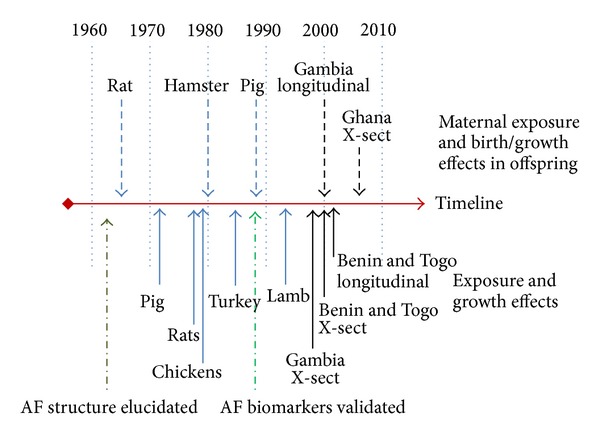
A timeline for the discovery and identification of aflatoxin and key studies in animals and humans on growth in relation to dosing or natural exposure, respectively. Human data only includes validated exposure biomarker driven studies; additional human studies published between 1989 and 2010 further support these observations (see text). Modified from [21, 22, 136].

The human epidemiological data presented provides compelling evidence for a role of aflatoxin in early life growth faltering. Animal data from experimental work and the veterinary literature [2–18]**   **strongly supports this epidemiological data. A timeline highlighting some of the critical animal data and human data where aflatoxin exposure was associated with growth is shown in Figure 5. In one such study a reduced weight gain in rats was observed in AFB1-treated compared to control animals, an effect partially restored by treating animals with a probiotic that reduced aflatoxin bioavailability [8]. Whilst the mechanism of aflatoxin induced growth faltering remains unclear, demonstration of improved infant growth following interventions that can restrict aflatoxin exposure in some of the high risk regions of the world is now critical. Understanding the mechanism of these effects would provide further evidence of causality of these events.

One putative mechanism is a direct toxicity of aflatoxin on the integrity of the intestine [137], and it is plausible that gastrointestinal bacterial infections, a major cause of growth faltering [129, 130, 138–143], and aflatoxins either additively or synergistically cause prolonged gastrointestinal enteropathy leading to poor nutritional uptake and nutrition retention, in regions where diets are frequently “nutritionally poor.” Cotoxicities of xenobiotics and infections are an increasingly emerging area of concern [144] in the etiology of chronic disease, and it is already established that aflatoxins and hepatitis B virus play a synergistic role in the high incidence of hepatocellular carcinoma in certain world regions [145–149]. In the context of growth faltering, a putative interaction between bacterial infection and aflatoxins is of particular concern as aflatoxins are also potent suppressers of the immune system [150], and in aflatoxin exposed Gambian children a reduced protection at mucus membranes in the form of significantly lower mean level of secretory IgA was observed when compared to nonexposed Gambian children [95]. Thus both the infection and aflatoxin may support a cycle of prolonged intestinal enteropathy. Additionally, given the essential role of the liver in overall homeostasis, aflatoxin induced liver toxicity per se may play an important role in observed growth faltering. Some of the liver damage may also result from increased levels of endotoxins penetrating an aflatoxin damaged gut lumen, entering the hepatic portal vein and subsequently additionally contributing to liver toxicity [151–153]. These putative mechanisms remain poorly examined to date. In terms of maternal exposure and growth faltering, one study in swine identified aflatoxin growth faltering in piglets following maternal exposure during the in utero period. Authors suggested that this may reflect changes in zinc bioavailability in the offspring [14].

It is perhaps worth trying to put these reported observations of aflatoxin on child growth into a somewhat more global context. It has been estimated that about 165 million children under the age of five years, predominantly in low-income countries, suffer from chronic undernutrition. These children are either stunted (HAZ < −2) or underweight (WAZ < −2). Poor growth in early life goes beyond just being a little shorter, but rather has significant consequences related to risks of illness and death in childhood and into adulthood. A recent meta-analysis through the Cochrane Group identified hygiene as a significant predictor of growth. Studies that were included in the review involved those that improved the quality of drinking water, introduced new or improved water supply or distribution, introduced the coverage and use of facilities designed to improve sanitation, or promoted hand washing after defecation [141]. This analysis included many thousands of children and revealed that improved hand hygiene in relation to defecation would significantly improve linear growth in children underfive years by, on average, 0.5 cm, that is, modestly improve stunting, though not affecting being underweight. This made international news headlines [154, 155]. In the five studies reported above on aflatoxins, differences between chronic low levels of aflatoxin exposure and chronic high levels of exposure are predicted to have greater adverse effects on both stunting and being underweight [98, 104].

One missing piece of the puzzle is that to date no single study has followed aflatoxin exposure through pregnancy and into the first 3–5 years of life. This could be valuable in terms of clearly establishing critical windows of exposure, understanding mechanisms of effect, understanding dose response relationships, and opportunities and timing of intervention. Given that growth faltering in poor rural settings in which aflatoxin is proposed to have an effect is a complex process associated with diet, infection, maternal health, and season of birth [132], establishing clear dose response effects of aflatoxin may not be trivial. One important public health message could simply be the reinforcement of the prolonging of breastfeeding in at risk populations. Whilst aflatoxins are transferred to breast milk, the levels are modest compared to that in weaning foods [98, 102–104] and through the weaning process aflatoxin biomarker levels follow a pattern from low to high as you move from exclusively breast fed to partially breast fed to fully weaned. Other intervention strategies that either reduce aflatoxin contamination of dietary staples [97] or affect uptake or biotransformation (see below) will also be important to protect maternal and postweaning phases of exposure. The development of sustainable targeted interventions should be a priority given the clear burden of exposure.

## 4. Potential Intervention Strategies

Intervention strategies to restrict human exposure to aflatoxins can be divided into two main activities: high tech agricultural methods and low tech research approaches. The first involves methods to restrict the growth of aflatoxin producing fungi. These include activities such as irrigation systems, genetically modified resistant crops, weather monitoring systems, rapid efficient harvesting, drying, and storage. Such approaches are expensive and thus only applicable to wealthier regions of the world. Such regions additionally have sufficient resources to discard and thus remove contaminated foods from human and animal food chains. In this review we will focus more on the low tech end of the spectrum with an emphasis on some of the higher risk regions, with the higher risk being due to (a) reliance of limited dietary staples, for example, maize and groundnuts; (b) predominant reliance on local or own grown staples; and (c) poor long-term (many months) storage of dietary staples.

The low tech approaches have broadly followed three main thought processes: (a) accept dietary contamination but given that aflatoxin requires bioactivation, modify either the activation or the detoxification process; (b) accept dietary contamination but given that the toxin has to cross from the gut lumen to the circulatory system, restrict that absorption; and (c) do not accept dietary contamination and implement simple postharvest approaches to restrict the contamination. Those methods discussed here were envisioned with a restriction of aflatoxin driven cancer in mind but are applicable here also.

### 4.1. Modification of Aflatoxin Biotransformation

Aflatoxins are biotransformed by phase one reactions to a mixture of metabolites with either lesser toxicity or greater toxicity. The reactive aflatoxin epoxides can cause damage to both DNA and proteins. For a given individual the amount of toxin ingested and the balance between epoxide formation and epoxide detoxification contribute to the burden of toxicity. The family of glutathione S-transferases are a critical detoxification system. Animal models have clearly demonstrated the utility of glutathione-S-transferase induction as chemoprevention against aflatoxin [156]. Chemoprevention has been demonstrated by a variety of chemically similar compounds, though oltipraz is one of the few that have moved into clinical trials to restrict aflatoxin.

Induction of glutathione-S-transferase is thought to be the dominant mechanism of oltipraz in the reducing aflatoxin-DNA adduct formation, aflatoxin-albumin formation, and aflatoxin driven hepatocarcinogenesis in animals [157–162]. This effect may be further modified by restrictions in phase 1 enzymes (CYP1A2 and CYP3A4) activity [163, 164]. Thus both the formation of the reactive aflatoxin-epoxide and its detoxification are enhanced by oltipraz. Oltipraz was subsequently successfully used in a clinical trial in China during 1995 to demonstrate proof of principle in populations naturally exposed through diet [165]. The study had three groups (control, low dose intervention, and high dose intervention) and included an intervention period (8 weeks) and a clear-out period (8 weeks). AF-albumin was used to assess the efficacy of the intervention, and by the second month the relative change on the high dose intervention, compared to the baseline, was significantly greater than in the placebo (*P* < 0.001), but no significant difference was observed for the low intervention compared to control [111]. Urinary AFM1 was also assessed as an exposure biomarker in this clinical trial, and the concentration was lower in both intervention groups compared to the control, though this only reached statistical significance for the higher oltipraz dose [166]. Finally the concentration of aflatoxin-mercapturic acid (AF-Ma) was measured; this is a putative though nonvalidated biomarker of aflatoxin detoxification; thus higher concentrations were predicted to indicate upregulation of glutathione-S-transferase. This biomeasure was higher in both intervention groups, though only significantly at the lower oltipraz dose [166]. These data support a role for aflatoxin chemoprevention via both a reduction in phase one enzyme activation systems and a gain of phase two detoxification.

The above approach while scientifically stimulating raises the issue of how to actually intervene in exposure, in poor settings, for millions of individuals using a drug based approach. The challenge may be better served using more natural components of the diet and notable contributions have been made by the work with broccoli sprouts and green tea polyphenols [167–175]. A number of cruciferous vegetables including broccoli are rich in glucosinolates; one, in particular, glucoraphanin, can be metabolized to sulphoraphane, an inducer of the phase two detoxification via upregulation of the glutathione-S-transferase family of enzymes. The enzyme responsible for the transformation of glucoraphanin, myrosinase, is found naturally both in the plant material (allowing enzyme and substrate to mix and interact during maceration) and within the gut microflora of animals including humans. Within a population there are significant variations in the capacity of the gut microflora to biotransform glucoraphanin to sulphoraphane [71] and thus for some individuals the quantity of myrosinase released from the broccoli may significantly impact the “apparent efficacy” of the approach to intervene in aflatoxin exposure. The first clinical trial [176] was conducted following successful demonstration of the efficacy in animals exposed to dietary aflatoxins [167, 168]. In the context of controlled clinical trials considerable effort was put into deciding on the best delivery approach for the broccoli such that all of the participants within the intervention arm received the same dose, in a manner that was compatible with a potentially “normal” dietary scenario. To this end a broccoli sprout infusion was prepared on a large scale, and intervention participants consumed the infusion during the study. The aflatoxin in the diet was based on the normal pattern of exposure that would occur from predominantly maize in this region; natural dietary exposures, and their restriction, are assessed within all the intervention studies discussed here. One limitation of this study was the loss of broccoli myrosinase enzymes due to the heat labile nature of proteins during infusion preparation. Individuals in one arm received the broccoli infusion over two weeks, while the other arm was a nonbroccoli control. Other cruciferous vegetables were excluded from the diet for both groups. Overall there was a modest nonstatistically significant reduction in urinary aflatoxin-N7-guanine when comparing broccoli infusion consumers to a control infusion group. However, within the infusion consumers the gut based myrosinase activity was additionally assessed, and an inverse relationship was observed between gut flora myrosinase activity and the aflatoxin biomarker (*P* < 0.001, *r* = 0.33). Thus to assess the efficacy of the intervention, both the amount of the intervention “dose within the broccoli” and the individual variations in conversion of that dose into the active ingredient need to be accounted for. Overall the approach was successful, though for better efficacy raw broccoli sprouts should be consumed to allow a greater formation of the active sulphoraphane.

This type of chemoprevention approach has important public health implications beyond that of aflatoxins. Many lipophilic carcinogens to which we are exposed will be metabolized by these enzymes and thus in general an increase in phase 2 detoxification pathways may significantly reduce toxicity. One good example that serves to highlight this potential protective effect was demonstrated using broccoli again in China, in which a demonstrated modulation of the concentration of several important metabolites of polyaromatic hydrocarbons (PAH) in urine was reported [71]; the PAH exposure was predicted to be from air-borne pollutants.

The other much discussed approach has been with green tea polyphenols; again in animals they have been demonstrated to alter aflatoxin metabolism, reduce the aflatoxin adduct burden, and reduce aflatoxin induced toxicity [169]. The first clinical trial was a randomized, double-blind, placebo-controlled clinical trial in Southern Guangxi, China [170]. The trial provided an interesting extension of the biomarker approach to assess efficacy and thus is particularly noteworthy. One of the difficulties in mycotoxin intervention trials in real world settings is clearly demonstrating the effectiveness of the intervention when the dietary levels of toxin exposure are highly variable amongst the study participants. By understanding the toxicokinetics of the exposure, in this case the aflatoxin contaminant, these authors investigated not only the concentration of the individual biomarkers, but also the ratio of detox/activation pathways when assessing the efficacy. The advantage here is that the “apparent efficacy” of the intervention will have less confounding based on the natural high variation in exposure. The study involved a low, a high, and a placebo control group and collected blood and urine at the outset, after one month and again at three months of the trial. Both intervention groups had a significant increase in the ratio of aflatoxin-mercapturate/aflatoxin M1, indicative of greater levels of detoxification. At one month the mean ratios were 2.2 ± 4.0, 22.1 ± 58.4, and 8.2 ± 15.9 and at 3 months they were 5.4 ± 6.9, 16.5 ± 22.9, and 12.5 ± 17.2, for control, low dose, and high dose interventions, respectively, (*P* < 0.05 at least) for all.

Other natural chemoprevention approaches include coumarin [177], coffee diterpenes [178], and bioflavonoids from kola seeds [179]. There has also been interest in the use of the group of compounds based on pentacyclic oleanane triterpenoids [138, 180].

### 4.2. Modulation in Bioavailability

An alternative approach is the use of compounds/materials that restrict the bioavailability of the toxin within the gut. This approach binds and shuttles a greater proportion of the ingested toxin through the gut such that it can be excreted directly by fecal elimination. In many regions within sub-Saharan Africa consumption of clays in low amounts is a regular and acceptable dietary activity [181]. After examining many different potential clay sorbents, a material was identified and tested which had high capacity to bind aflatoxins, and critically in animals models and during early clinical trials, the use of dietary clay led to no adverse observable health effects or modulation of critical mineral or vitamin levels [181–183]. The aflatoxin binder is known as NovaSil clay. In numerous animal systems significant reductions in aflatoxicosis have been reported [181]. The first clinical trial was conducted in Ghana, West Africa, and in a similar approach to those discussed for chemoprevention in China, the trial was double blind and placebo controlled, involving individuals naturally exposed through diet. The trial involved 177 individuals who were preselected because they were positive for serum AF-albumin; these participants were randomly assigned to a high dose (3.0 g clay/day), low dose (1.5 g clay/day), or placebo control group, for 3 months [184]. No differences were apparent between control and intervention groups for hematology, liver or kidney function, serum biochemistry, or serum nutrients [184]. The efficacy of the intervention was assessed using both serum AF-albumin and urinary AFM1 as exposure biomarkers. The urinary AFM1 concentrations varied significantly by individual (range 0.04–13,298 pg AFM1/mg urinary creatinine (pg/mg)), but data were less variable for AF-albumin. At baseline there were no significant differences between the three groups in the median concentration of AFM1, nor the mean concentration of AF-albumin. There were no significant differences in urinary AFM1 between the low dose intervention and the control at either time point. There was no significant difference for the high dose at the 3-month time point, but a 58% reduction in AFM1 was observed at the 1-month time point. One concern therefore is why the effect was not observed at both time points and perhaps highlights the complexity of demonstrating efficacy of an intervention approach when the daily exposure to toxin is highly variable within even a relatively similar study population. For AF-albumin modest reductions of 3.2% and 6.4%, for low and high doses, respectively, were observed at the 1-month time point, albeit nonsignificant for both, whilst statistically significant reductions by both treatments of about 25% were observed at the 3-month period. One month after intervention no statistically significant differences were observed [110]. The delay before a statistically significant effect on AF-albumin adduct level was observed, again probably reflecting the longer term nature of the albumin biomarker. Overall, the combined biomarker measures suggest the potential effectiveness of NovaSil clay for restricting aflatoxin uptake. Given that the use of clay particles at a low dose is acceptable in many at risk populations, this approach could be well tolerated. This approach was recently reported to have effectively reduced AFM1 biomarkers in Ghanaians [185]. Philips and colleagues are undertaking extensive surveys within West Africa of naturally occurring clays, such that the method is not continually reliant on a more developed country for the clay, a critical requirement if this approach is to become applied more widely over extended time frames. To date it is not apparent that such a material has been located. Where monitoring at a local level is economically feasible the use of the clay may have particular utility, and if acute poisoning incidence could be predicted through weather/drought monitoring and through advanced prediction of food scarcity, then this material could also provide essential temporary relief in aid programs where acute toxicity may also be an issue, as observed in recent years in Kenya [186–188].

There is additionally a perhaps more “usual” dietary approach with similar outcomes in terms of aflatoxin restriction [189–191]. Chlorophyllin (CHLN) and chlorophyll (CHLL) provide a particularly interesting approach to aflatoxin intervention. CHLN is a synthetic derivative of CHLL that has been demonstrated to significantly reduce liver aflatoxin-DNA adduct formation in trout treated with AFB1 [171] and to similarly reduce liver tumor incidence [189]. The mechanism of action is via the planar nature of both the chemical species and the ability for sufficient complex formation in aqueous solution such as that in the gastrointestinal tract; uptake of the aflatoxin from the gut is significantly reduced, and therefore systemic exposure lessened [172, 191]. The first clinical trial involving 180 volunteers took place in Qidong, China, in which the efficacy of the intervention was assessed using urinary AF-N7-Gua. The study design involved three months of thrice daily ingestion of CHLN in the intervention group and a placebo for the control and the natural exposure of the population to aflatoxin through diet. At the 3-month time point a significant (*P* < 0.05) reduction in median urinary AF-N7-Gua in the intervention (0.09 pg/mg creatinine) compared to the control (0.20 pg/mg) was observed [173]. Thus the use of CHLN as a chemointervention drug to restrict aflatoxin appears plausible. What is perhaps more interesting is to understand whether the naturally occurring form, that is, CHLL, has similar properties, as dietary consumption of moderate to high levels of green leafy vegetables would provide a similar dose of CHLL as the above trial with CHLN [174]. In rodents CHLL has been demonstrated to reduce both the levels of aflatoxin exposure biomarkers, notably serum AF-albumin and urinary AF-N7-Gua, and the levels of hepatic toxicity, notably hepatic aflatoxin-DNA adduct level and volume occupied by GST placental form positive foci in the liver [174]. A toxicokinetics study in which four individuals were dosed with minute quantities of radiolabeled AFB1 in the absence or presence of CHLL also revealed that CHLL reduced bioavailability of the toxin [175]. The use of radiolabeled toxin allows the exquisite sensitivity of accelerator mass spectrometry to be used for quantitation [113], a technique able to accurately measure attomole quantities of analyte, such that human dosing studies at “safe levels” can be conducted to better understand the toxicodynamics and kinetics for chemical exposures of significant public health concern. Whilst this human study was limited in size, these data support the running of a clinical trial to restrict natural exposure to aflatoxins using a natural and frequently available product, that is, green leafy vegetables.

A third option that has been suggested comes from observations that a number of bacterial strains commonly used in milk or yogurt based foods are able to bind aflatoxins and some other mycotoxins [8, 55, 137, 192–195]. *Lactobacillus rhamnosus* strain GG (GG) and strain LC705 were particularly efficient in binding of aflatoxins [192, 193, 196]. It is thought that these products may provide protection by shuttling bound aflatoxins through the gastrointestinal tract and thus reduce systemic uptake from the gut. It has further been postulated that regular consumption may lead to significant gut colonization such that, once achieved, continued consumption of the probiotic may not be necessary. For the latter, it is not as yet clear if this is possible, and if some colonization did occur, it is not clear whether the probiotics would colonize in a region of the gut that would provide significant protection, that is, the region of colonization that was relevant to where most aflatoxins are absorbed.

In vitro studies in caco-2 cells, ex vivo studies in the intestinal lumen of chicks, and in vivo studies in rats have demonstrated that probiotics are able to bind aflatoxin and to reduce toxicity [137, 194, 197], including the partial restoration of aflatoxin induced growth faltering [8]. A double-blind, placebo-controlled clinical trial of 90 healthy males from Guangzhou, China, assessed the efficacy of the intervention in a population naturally at risk from aflatoxin exposure through diet [198, 199]. Individuals were randomly assigned to either the intervention group (*L. rhamnosus *LC705 and *Propionibacterium freudenreichii *subsp. *shermanii*) or a placebo control group (cellulose), for five weeks. Morning urine samples were collected at baseline, week 3, week 5, and week 10, with the latter being five weeks after intervention to measure AF-N7-Gua. A nonsignificant increase in the frequency of nondetects was observed for the intervention group, whilst significant reductions in mean urinary AF-N7-Gua of 36% and 55%, for weeks 3 and 5, respectively, were observed, and overall *P* value was 0.005. No differences were observed at baseline or five weeks after the intervention ceased, suggesting that within this study constraints and time colonization to any significant extent had not occurred or had not occurred in a region where restriction of aflatoxin uptake was physiologically important.

Probiotic strains of *Lactobacillus casei*, *Lactobacillus plantarum,* and *Enterococcus faecium* also may provide protection against aflatoxins [200–202]. The ability of any particular probiotic strain to limit aflatoxin bioavailability probably depends on the contaminated food consumed [203], so multiple probiotic strains may be needed to provide effective protection. The use of probiotics that are capable of detoxifying mycotoxins within food processing activities may have both local and industrial benefits but is beyond the scope of this review. Probiotics in the diet may of course provide additional benefits to gastrohepatic health beyond reducing aflatoxin bioavailability. Probiotic use is probably not a useful approach to mitigate aflatoxin exposure in some of the poorest, rural settings with chronic exposures such as in some parts of sub-Saharan Africa. However, it is plausible that probiotics may be a useful intervention in middle income populations, with moderate frequencies and levels of aflatoxin exposures, for example, Thailand, Brazil, and Egypt, where milk products may be used as a vehicle to supply the probiotic.

### 4.3. Postharvest Intervention

An alternative approach is to develop integrated educational community based programs that aim to improve postharvest drying and long-term storage of at risk foods in rural subsistence farm settings, thus restricting contamination and growth of *Aspergillus* fungi and lowering aflatoxin contamination. One intervention study of this nature was conducted in the lower Kindia region of Guinea [97]. Farms from 20 villages were included: ten control villages (*n* = 30 participants per village) and ten villages (*n* = 30 participants per village) where a package of postharvest measures to restrict aflatoxin contamination of the groundnut crop were undertaken. These include rapid collection of the nuts from the field at harvest, drying of the nuts, and storage of the nuts in jute sacks (breathable bags) which were additionally stored raised off of the ground, with an insecticide sprinkled under the stored food. The control villages simply did their normal postharvest practices. The efficacy of the study was based on the entire package and was assessed using AF-albumin adducts immediately after harvest and at 3 months and 5 months afterharvest. In control villages AF-albumin levels indicated the expected trend of increasing exposure following prolonged storage. The mean AF-albumin level significantly (*P* < 0.001) increased by approximately 250% (geometric mean 5.5 pg/mg [95% CI 4.7–6.1] immediately after harvest to 18.7 pg/mg [17.0–20.6] 5 months later). There was no difference in the AF-albumin between the control and intervention at the time of harvest, but five months into the intervention the mean AF-albumin had only increased by only 11%, a significant (*P* < 0.001) improvement compared to the 250% increase of the control group. Thus this low-technology approach provides a significant protection against aflatoxin exposure for the subsistence farmer in sub-Saharan Africa. It will be important to expand this study trial to other regions and to the maize crop.

## 5. Coexposures to Other Mycotoxins

It has been evident for some time that food items are not contaminated with single toxins; thus exposures at any given time point and throughout life are multiple and varied. Despite this, the effects of coexposures are at best mostly poorly understood. Within the mycotoxin arena it has been suspected for some time that the trichothecene mycotoxin deoxynivalenol may have adverse effects on the immune system and growth, an observation based on the animal data [21, 150]. In maize consumers in tropical regions the fumonisins are another important family of mycotoxins. In a recent survey of infants from Tanzania estimates of fumonisin intake were made using FB contamination levels of foods and amounts consumed. At 12 months of age infants were significantly shorter by 1.3 cm and 328 g lighter when their estimated intakes of fumonisins exceeded the provisional maximum tolerable daily intake (2 *μ*g/kg bw/day) compared to those with lower estimated FB intake [204]. No such data are available for deoxynivalenol at this time.

In recent years several groups have investigated putative exposure biomarkers for these two toxins, in an attempt to better improve exposure assessment. For deoxynivalenol the combined measure of urinary deoxynivalenol and its glucuronide (total deoxynivalenol) was suggested around 10 years ago [205]. The analytical tools were further developed [100, 101], and subsequently a strong quantitative relationship between this urinary measure and the deoxynivalenol intake was demonstrated, (adjusted *r* ^2^ = 0.83, *P* < 0.001) [206]. Thus it appears that the variation in urinary total deoxynivalenol is well explained by the intake. It was notable that whilst a statistically significant relationship was observed between the urinary measure and typical cereal intake (adjusted *r* ^2^ = 0.24, *P* < 0.001), more recent cereal intake (*r* ^2^ = 0.27, *P* < 0.001), or estimated DON intake based on the cereal consumption pattern (*r* ^2^ = 0.22, *P* = 0.02), the variation in urinary deoxynivalenol was poorly to modestly explained by simple assessment of the diet [100, 207, 208]. The strength for this exposure biomarker thus relies on the demonstrated strong quantitative measure of actual intake versus the urinary measure [206]. In addition, an intervention to restrict wheat consumption showed a significant 11-fold reduction in mean biomarker levels in the preintervention [101], geometric mean 7.2 ng DON/mg creatinine (95% CI: 4.9–10.5 ng/mg), to a level following four days of dietary wheat restriction of 0.6 ng per mg (95% CI: 0.4–0.9 ng/mg).

The development of a fumonisin exposure biomarker has also been undertaken. Most recent work has focused on measuring urinary fumonisin B1 [25, 209–211]. Urinary fumonisin B1 was frequently observed in individuals reliant on maize as a dietary staple, in regions with demonstrated or suspected fumonisin contamination of the maize. A significant relationship between tortilla consumption and the urinary measure (FB1 pg/mL urine or FB1 pg/mg creatinine) was reported for a survey of 75 Mexican women, preselected to represent low, medium, and high consumption, based on consumption data from a larger survey (*P* < 0.001). In a separate study a modest but significant (*r* ^2^ = 0.25, *P* < 0.01) relationship was observed between measures of dietary fumonisin (mean of two-day intake) and the urinary measure assessed in pg/mg (mean of two-day measure) for 22 South African women during their normal diet and following an intervention [209]. The *r* ^2^ for the baseline only phase was 0.31. In an intervention that reduced the food contamination levels of fumonisin in maize that these women were consuming, a nonsignificant (*P* > 0.05) reduction in the urinary biomeasure from baseline levels (geometric mean 470 pg/mg: 95% CI 295, 750 pg/mg) compared to those after intervention (geometric mean 279 pg/mg: 202, 386 pg/mg) was observed [209]. In another study in China, differences in urinary fumonisin were observed between a predicted high and a predicted low exposure region; the median urinary-free FB1 level in Huaian subjects, *n* = 43 (3.9 ng/mg; range nondetect–253.6 ng/mg) versus Fusui subjects, *n* = 34 (0.4 ng/mg; range nondetect–3.7 ng/mg), (*P* < 0.01) [210]. These authors however also state that overall the relationship between fumonisin intake and urinary fumonisin was not significant (*P* > 0.05).

A controlled dosing study with fumonisin B1 has been conducted in healthy US residents, *n* = 10 [211]. In this study the average intake of a fixed quantity of FB over several days was compared with urinary excretion. The major observation was that on average about 0.5% of the excreted fumonisin was transferred to urine, about 7 times greater than that estimated for the South Africa study [209]. The US-based study also reported a wide range in the amount transferred. This wide variation in the excretion kinetics in a very controlled situation raises some concern on the use of this exposure biomarker in epidemiological studies in comparison, for example, with those using aflatoxin biomarkers. Clearly some relationships exist, but the lack of overall strength in the relationships reported from studies to date is suggestive that additional care will be required in sample size calculations for epidemiological studies that use this measure. Nonetheless, aflatoxin and fumonisins are likely to frequently cooccur and it will be important to understand the effects of such events [119]. Multimycotoxin methods may also assist more rapid assessment of multiple exposures including those from aflatoxins, fumonisins, and deoxynivalenol [212–214].

Both of these novel measures are urinary markers and thus will reflect only recent exposure to the toxins. It will be important to assess the temporal nature of these measures in individuals. By contrast AF-albumin represents exposure over an extended period, though the ideal would be to have tools that indicate exposures over years. Such tools remain to be developed.

## 6. Conclusion

Aflatoxins are highly potent secondary metabolites that contaminate dietary staples in tropical regions, regions where growth faltering often has a significant burden on life expectancy. Where diets are frequently contaminated, epidemiological studies require biological measures to better understand exposures. For aflatoxins several “biomeasures” have undergone vigorous validation processes and are classified as exposure biomarkers. One such exposure biomarker, AF-albumin, reflects an integrated measure of exposure over several months. In several tropical regions of the world AF-albumin is detected in >95 of all tested samples once the child is weaned, and when detected, levels are often high. Several key epidemiological studies show strong associations between this biomarker and reduced birth weight or growth faltering, associations that remain after adjustment for confounders. When one considers that worldwide 40% of the 11 million deaths in children aged less than 5 years old occur in sub-Saharan Africa [139] and that approximately half of the deaths linked to infectious diseases in sub-Saharan African children point to undernutrition and slowed growth as an underlying cause, the urgent need for further research into the effect of these food contaminants on public health becomes self-evident. Since mycotoxin-contaminated foods constitute a large portion of daily dietary intake for many of the world's developing nations, assessments of mycotoxin exposure are essential, as is the need for clarification of the biological mechanisms involved. Such understanding of the health risks may lead to targeted, affordable, and sustainable methods being established to restrict such exposures among those at highest risk and to reduce the overall burden of mycotoxin driven chronic disease. Understanding the potential role of other mycotoxins and how to intervene may also be critical. Numerous intervention studies are possible based on modifying exposure through natural chemopreventative activities; however, none will completely eliminate exposure. Education packages that improve drying and storage have proved highly successful in a postharvest intervention trial aimed at the storage of groundnuts in Guinea [96]. It will be valuable to extend intervention education such that reduction in food contamination and chemopreventative approaches combined may restrict the burden of chronic aflatoxin driven diseases.

## Figures and Tables

**Table 1:** AF-albumin adducts concentration distribution from studies 1–4 on assessing growth and data from Canada and the USA. Data presented are the percentage within each study within one of the pg/mg concentration groups (modified from [21, 22, 95, 98]: [102–104]; [120]).

			AF-albumin distribution (pg/mg)						
			<5	<25	<50	<100	<200	<500	>500
	Canada	Adults (*n* = 200)	100	0	0	0	0	0	0

	USA	Adults (*n* = 10,000)	100	0	0	0	0	0	0

Study 1	Gambia	6–9 years (*n* = 478)	8	45	28	13	4	2	0

Study 2	Benin and Togo	<2 years (*n* = 152)	5	58	18	11	6	2	0
		<3 years (*n* = 135)	2	31	27	21	11	4	2
		>3 years (*n* = 192)	2	24	38	15	14	6	1

Study 3	Gambia	Maternal (*n* = 113)	0	30	35	20	12	5	0
		Cord blood (*n* = 109)	54	41	1	2	2	0	0
		Week 16 (*n* = 110)	89	9	2	0	0	0	0
		Week 52 (*n* = 113)	7	49	16	11	9	9	0

Study 4	Benin and Togo	16–38 months (*n* = 197)	2	34	22	18	15	7	1
		21–43 months (*n* = 194)	3	29	25	25	12	6	1
		24–46 months (*n* = 193)	1	16	17	18	22	18	8
